# An Open-Source QAM MODEM for Visible Light Communication in FPGA for Real-Time Applications

**DOI:** 10.3390/s26030992

**Published:** 2026-02-03

**Authors:** Stefano Ricci

**Affiliations:** Information Engineering Department, University of Florence, St. S.Marta No. 3, 50129 Florence, Italy; stefano.ricci@unifi.it

**Keywords:** Quadrature Amplitude Modulation (QAM), Visible Light Communications (VLC), FPGA, real-time, low-latency, VHDL, open-source code

## Abstract

Visible Light Communication (VLC) is a transformative paradigm poised to revolutionize the automotive and numerous other sectors. As the demand for high data rates and low latency applications grows, the limited bandwidth of standard white LED-based lamps—typically restricted to a few MHz—presents a significant bottleneck. While high-order modulation schemes like Quadrature Amplitude Modulation (QAM) offer superior spectral efficiency, their computational complexity often hinders real-time implementation. Consequently, the existing literature lacks experimental validation of low-latency real-time VLC links. This work addresses this challenge by proposing a modified algorithm that is implemented in a resource-efficient QAM modulator/demodulator (MODEM) for an FPGA. The algorithm includes the synchronization loop. The proposed MODEM is available as open-source code and provides a scalable foundation for researchers to explore low-latency real-time VLC links. Experimental results demonstrate successful 2, 4, and 6 Mb/s links using 4-, 16-, and 64-QAM constellations, respectively, over a white-phosphor-power LED. We measured a latency of less than 1.3 μs.

## 1. Introduction

Visible Light Communication (VLC) represents a novel emerging method for transferring information using visible light [1,2]. In VLC, the information is modulated by varying the intensity of an illumination source, using frequencies that are imperceptible to the human eye but are detectable by a photoreceiver. According to this new paradigm, an illumination source behaves simultaneously as a transmitter. A significant boost to this technology was given by the recent substitution of incandescent and fluorescent lamps with Light Emitting Diodes (LEDs) [3], which are suitable for light modulation.

With its localized communication range, use of the underutilized optical spectrum rather than the saturated RF band, immunity to RX interference, and compatibility with existing LED lamps [4], VLC addresses several fields, but it is uniquely positioned to face the challenges present in automotive applications [5]. The versatility of VLC fosters a diverse array of use cases that are moving the world towards a new transportation system, such as Vehicle-to-Vehicle (V2V) networks [6], intelligent traffic control [7], Cooperative Intelligent Transportation Systems (C-ITS) [8], and many others.

The simplest way to modulate light for communication is to use the On–Off Keying (OOK) protocol, where light is rapidly switched between two different intensity levels. OOK, regulated by standards like the IEEE 802.15.7 [9], can be implemented through very simple transmitters (TX) [10], driven by microcontrollers for low rates at high distances [11] or by Field Programmable Gate Array (FPGA) for higher rates at shorter distances [12].

Most of the aforementioned applications require increasingly higher data rates [13], but, unfortunately, the useful bandwidth available from a white-phosphor LED, like those employed in automotive headlights, is limited to a few MHz [14]. The bandwidth efficiency of OOK is very low, in the order of 1 bit/s/Hz; thus, OOK does not represent an optimal exploitation of the limited LED bandwidth [15]. Quadrature amplitude modulation (QAM) performs significantly better, but its real-time implementation needs much higher computational power [16] sourced from high-end processors or FPGAs [17]. In the literature, many works have exploited QAM, but most of them are limited to theoretical studies based on simulations; when experiments are present, they are often performed through high-end bench instrumentation, like signal generators for transmitting and oscilloscopes for acquiring. In these works, the processing for modulating and demodulating the QAM signals is entirely performed offline in Matlab 2025 (MathWorks, Natick, MA, USA) or other similar tools. Most of all, the synchronization of the receiver to the frequency and phase of the QAM transmitter carrier [16], necessary in a real application, is often completely ignored in favor of other aspects.

In summary, despite the large number of papers on QAM, very few of them verify the proposed theory in complete real-time low-latency experiments. This situation has produced a gap in the field of VLC research, which is even more pronounced when considering the need for testing low-latency links, whose importance is paramount, among others, for safety-critical vehicle applications [18].

### 1.1. Related Works

Here, we present a brief review of the relevant papers that address a real-time VLC link based on QAM or QAM implementation in an FPGA or embedded processor. In [19], the authors present a real-time VLC with a physical layer (PHY) design that reduces the inter-symbol interference (ISI). Processing is performed in an FPGA. In [20], a Software-Defined Radio (SDR) hardware connected to LabVIEW (National Instruments, Austin, TX, USA) is used to test a specific adaptive equalization method that enables high-order QAM in a quasi-real-time environment. In [21], an elementary QAM modulator is implemented in an FPGA and verified through an oscilloscope. In [22], an adaptive equalizer for QAM is demonstrated, and an FPGA implementation is proposed. However, the results are limited to FPGA simulations. Meanwhile, the authors of [23] present a Carrier Recovery Loop for a 16-QAM modulator/demodulator (MODEM) and its FPGA implementation. In [24], a 16-QAM VLC link is demonstrated at low data rate. Here, the processing is performed in microcontrollers rather than FPGAs. In [25], the FPGA implementation of an adaptive equalizer and carrier recovery loop for a 50 Mbps 16-QAM receiver is presented. More about the aforementioned implementations is reported in Section 4.3, where they are compared to the method proposed in this work.

### 1.2. Our Contribution

As noted before, the papers that propose real-time QAM implementations in a VLC link are limited. In this work, we try to reduce this gap by presenting a novel QAM algorithm and its real-time and low-latency FPGA implementation. The proposed algorithm differs from the standard method present in books and papers, since it presents mathematical solutions that allow a notable resource saving when implemented in FPGA. The result is a low-complexity low-latency complete QAM MODEM, intended to serve as a versatile starting point for the community to deploy and refine custom real-time optimization techniques. For this reason, the FPGA code is developed completely in Very High Speed Integrated Circuit (VHSIC) Hardware Description Language (VHDL) [26] for inter-platform compatibility and is available in open-source format for the community (see Supplementary Materials note at the end of the paper).

The paper proceeds as follows: Section 2 establishes the mathematical foundation of a standard QAM MODEM, concluding with an analysis of the challenges inherent in FPGA implementation. These constraints serve as the rationale for the modifications proposed in the new algorithm described in Section 3, together with the design trade-offs and compromises involved. The FPGA implementation and its characterization under finite-precision arithmetic are presented in Section 4. Finally, Section 5 validates the design through a proof-of-concept experiment, demonstrating the QAM MODEM’s performance within a real-time VLC link based on a phosphorous white LED.

## 2. The QAM

### 2.1. Fundamentals of QAM

Here, we provide a brief summary of QAM mathematics for the reader’s convenience. A complete description can be found in books, for example [27].

#### 2.1.1. The Transmitter

**Figure 1:**
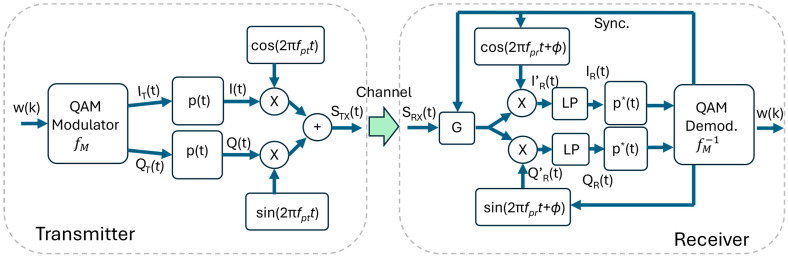
Schematics of a basic QAM transmitter (**left**) and receiver (**right**).

The basic QAM transmitter is sketched on the left side of Figure 1. Let us consider a QAM modulation with a constellation composed by M = 2^N^ complex symbols and a sequence of word w(k), each composed by N-bit. A QAM modulation function $f_{M}$ associates each word w(k) to one of the M symbols, generating the symbol sequence $s(k)=I(k)+\sqrt{-1}Q(k)=f_{M}(w(k))$. To the time-discrete sequence w(k), we associate the corresponding time-continuous functions $w_{T}(t)$, and, correspondingly, we have $s_{T}(t)=I_{T}(t)+\sqrt{-1}Q_{T}(t)=f_{M}(w_{T}(t))$. The function $w_{T}(t)$ can be formalized as

(1) $$w_{T}(t)=\sum_{i}w(i)\delta (t-i\cdot T_{s}-T_{s}/2)$$

where $T_{s}$ is the symbol time. This function is composed of a sequence of Dirac pulses δ(t) spaced by $T_{s}$ intervals and centered in the $T_{s}$ slot. At the output of the modulator (see Figure 1, left), we have the pulse sequences $I_{T}(t)\;$ and $Q_{T}(t)$, which are filtered by the pulse-shaping function with impulse response $p_{T}(t)$. The filtering produces a pulse with a limited bandwidth and reduces the ISI [28]. We note that the typical pulse-shaping functions are symmetric with respect to ${t=T}_{s}/2$, i.e., $p_{T}(T_{s}/2-t)=p_{T}(T_{s}/2+t)$. We will use this result later.

A quadrature modulator upconverts the data to a carrier of frequency $f_{pt}$:

(2) $$S_{TX}(t)\;=\left[I_{T}(t)*p(t)\right]\cdot cos\left(2\pi \cdot f_{pt}\cdot t\right)+\left[Q_{T}(t)*p(t)\right]\cdot sin\left(2\pi \cdot f_{pt}\cdot t\right)$$

Here, ‘∗’ represents the linear convolution. Finally (but not shown in Figure 1), $S_{TX}(t)$ is amplified and applied to a LED through a bias-tee that sources the Direct-Current (DC) necessary to maintain the mean current in the LED.

#### 2.1.2. The Receiver

Let us now refer to the right-side of Figure 1. If we assume a channel with a constant attenuation *A* in the bandwidth of interest, on the RX side, we have $S_{RX}(t)={AS}_{TX}(t)$. The signal $S_{RX}(t)$ is amplified by a factor *G* and then down-converted by a quadrature demodulator working at frequency $f_{pr}$, which nominally coincides with $f_{pt}$:

(3) $${I\prime }_{R}\left(t\right)=G\left[I_{T}\left(t\right)*p\left(t\right)\right]\cdot cos\left(2\pi f_{pt}t\right)cos\left(2\pi f_{rt}t+\Phi \right)+\;+G\left[Q_{T}(t)*p(t)\right]\cdot sin\left(2\pi f_{pt}t\right)cos\left(2\pi f_{rt}t+\Phi \right)\;{Q\prime }_{R}\left(t\right)=G\left[I_{T}\left(t\right)*p\left(t\right)\right]\cdot cos\left(2\pi f_{pt}t\right)sin\left(2\pi f_{rt}t+\Phi \right)+\;+G\left[Q_{T}(t)*p(t)\right]\cdot sin\left(2\pi f_{pt}t\right)sin\left(2\pi f_{rt}t+\Phi \right)$$

Equation (3) can be rewritten as

(4) $${I\prime }_{R}\left(t\right)=G\left[I_{T}\left(t\right)*p\left(t\right)\right]\cdot \frac{1}{2}\left[cos\left(2\pi \left(f_{pt}-f_{rt}\right)t-\Phi \right)+cos\left(2\pi \left(f_{pt}+f_{rt}\right)t+\Phi \right)\right]+\;+G\left[Q_{T}(t)*p(t)\right]\cdot \frac{1}{2}\left[sin\left(2\pi \left(f_{pt}-f_{rt}\right)t-\Phi \right)+sin\left(2\pi \left(f_{pt}+f_{rt}\right)t+\Phi \right)\right]\;{Q\prime }_{R}\left(t\right)=G\left[T\left(t\right)*p\left(t\right)\right]\cdot \frac{1}{2}\left[sin\left(2\pi \left(f_{pt}-f_{rt}\right)t-\Phi \right)+sin\left(2\pi \left(f_{pt}+f_{rt}\right)t+\Phi \right)\right]+\;+G\left[Q_{T}(t)*p(t)\right]\cdot \frac{1}{2}\left[cos\left(2\pi \left(f_{pt}-f_{rt}\right)t-\Phi \right)+cos\left(2\pi \left(f_{pt}+f_{rt}\right)t+\Phi \right)\right]$$

After a low-pass filter eliminates the components at $f_{pt}+f_{rt}$ frequency, we have

(5) $$I_{R}\left(t\right)=G\left[I_{T}\left(t\right)*p\left(t\right)\right]\cdot \frac{1}{2}\left[cos\left(2\pi \left(f_{pt}-f_{rt}\right)t-\Phi \right)\;\right]+\;+G\left[Q_{T}(t)*p(t)\right]\cdot \frac{1}{2}\left[sin\left(2\pi \left(f_{pt}-f_{rt}\right)t-\Phi \right)\right]\;Q_{R}\left(t\right)=G\left[I_{T}\left(t\right)*p\left(t\right)\right]\cdot \frac{1}{2}sin\left(2\pi \left(f_{pt}-f_{rt}\right)t-\Phi \right)+\;+G\left[Q_{T}(t)*p(t)\right]\cdot \frac{1}{2}cos\left(2\pi \left(f_{pt}-f_{rt}\right)t-\Phi \right)$$

Assuming that the receiver is synchronized to the transmitter (more on this below), we have $f_{pt}=f_{rt}$, and Φ=0; so, (5) is simplified as

(6) $$I_{R}\left(t\right)=\frac{G}{2}\left[I_{T}\left(t\right)*p\left(t\right)\right]\;Q_{R}\left(t\right)=\frac{G}{2}\left[Q_{T}(t)*p(t)\right]$$

These signals are optionally filtered by the filter $p^{*}(t)$, which is typically matched to the filter p(t) used in transmission. The filter output, down-sampled at rate $T_{s}$, represents the coordinates of the received vector. Finally, the QAM demodulator maps back the received vectors to the constellation points and recovers the digital words sequence.

#### 2.1.3. Synchronization

Like we mentioned before, the receiver must be synchronized to the transmitter. Two conditions are required for the communication link to work: (1) the receiver and transmitter oscillators must have the same frequency and phase; (2) the amplitude of the signal should be tailored so that the points in the TX and RX constellation match. When the oscillators’ frequency differs, the received constellation rotates at an angular velocity, which is proportional to the frequency difference. When the frequency is locked, but a phase difference is still present, the received constellation presents a fixed rotation. If the amplitude is not correct, the received constellation is a scaled replica of the original. In all cases, the reception is hampered.

The transmitter and receiver have local oscillators with the same nominal frequency, but unavoidable inaccuracies result in a frequency difference. To give an idea of the problem, a quartz oscillator has a typical accuracy of ±10 ppm; so, we can expect between the transmitter and receiver a frequency lag up to 20 ppm, which corresponds to a lag of a full symbol every 50 k. An example of the problems caused by the shift of TX and RX oscillators is reported in [29]. On the other hand, the difference in phase and amplitude depends on random factors; so, the receiver must compensate dynamically for phase and amplitude variations.

A wide range of synchronization methods have been studied: a review is reported in the book [30].

### 2.2. Limits of the Standard QAM MODEM

The implementation of the standard QAM MODEM in FPGA requires a non-trivial effort in terms of FPGA resources, clock frequencies, and ultimately, in cost. For example, a typical pulse-shaping function spans 4–10 samples, and with an oversampling rate of 10 samples per symbol, the resulting filter requires 40–100 taps. This high complexity extends to the receiver side, where both the low-pass and matched filters demand similar computational intensity. Synchronization algorithms further strain FPGA resources: they necessitate hardware-intensive operators such as square roots, divisions, and arctangents (e.g., CORDIC blocks), which occupy substantial logic area in FPGA [31].

In the next section, our solution to simplify part of this problem is described.

## 3. The Proposed Approach to QAM

The proposed QAM MODEM is designed with the goal of reducing as far as possible the FPGA resources needed for its implementation. This goal is pursued mainly through the three actions highlighted in Table 1, which will be detailed in this section.

Starting with the constraints outlined in N. 1 of Table 1, which apply to both the transmitter and receiver, we establish a synchronous relationship between the system’s timing parameters. Specifically, the carrier period is defined as an integer multiple of the sampling interval, and the symbol duration is defined as an integer multiple of the carrier period. Formally, these relationships are expressed as

(7) $$\frac{1}{f_{pt}}=k_{a}T_{c}\;T_{S}=k_{b}\frac{1}{f_{pt}}=k_{b}k_{a}T_{c}{=N_{s}T}_{c},$$

where ${T_{c}=1/f}_{c}$ is the sampling time, and $k_{a}$ and $k_{b}$ are natural numbers. It should be noted that the symbol is represented by $N_{s}=k_{b}k_{a}$ digital samples.

From now on, we will use the index i for locating the *i*-th sample among the $N_{s}$ samples that belong to a symbol ($0\leq i<N_{s}-1$), and index *k* for tracing a symbol in its sequence *s*(*k*). To clarify further, the samples i are transmitted at rate $f_{c}$, while for every $N_{s}$ samples, a new symbol *k* is issued.

### 3.1. The Transmitter

We substitute into (2) $f_{pt}=k_{b}/N_{s}$ and $t=iT_{c}$. We have

(8) $$S_{TX}\left(iT_{c}\right)=\left[I_{T}\left(iT_{c}\right)*p_{T}\left(iT_{c}\right)\right]\cdot cos\left(2\pi \frac{k_{b}}{N_{s}}i\right)+\left[Q_{T}(iT_{c})*p_{T}\left(iT_{c}\right)\right]\cdot sin\left(2\pi \frac{k_{b}}{N_{s}}i\right)$$

In practical implementation, the pulse-shaping function $p_{T}(t)$, sampled at $t=i/f_{c}$, must have a finite pulse response $p\left(i\right)$. According to N. 2 in Table 1, $p\left(i\right)$ is restricted to the same duration $T_{S}$ of the symbol; thus, p(i) has $N_{s}$ samples, like the symbol itself. Given the aforementioned constraints, we have

(9) $$I_{T}\left(iT_{c}\right)*p_{T}\left(iT_{c}\right)=I(k)\cdot p\left(i\right);\;Q_{T}(iT_{c})*p_{T}\left(iT_{c}\right)=Q(k)\cdot p\left(i\right)$$

Combining these elements and accounting for the sample index, we obtain the following expression

(10) $$S_{out}\left(k,i\right)=I(k)\cdot Tab\_pc\left(i\right)+{Qa}_{T}\left(k,i\right)=Q(k)\cdot Tab\_ps(i);\;0\leq i<N_{s}$$

where

(11) $${Tab}_{c}\left(i\right)=p\left(i\right)\cdot cos\left(2\pi \frac{k_{b}}{N_{s}}i\right);{Tab}_{s}(i)=p\left(i\right)\cdot sin\left(2\pi \frac{k_{b}}{N_{s}}i\right);\;0\leq i<N_{s}$$

**Figure 2:**
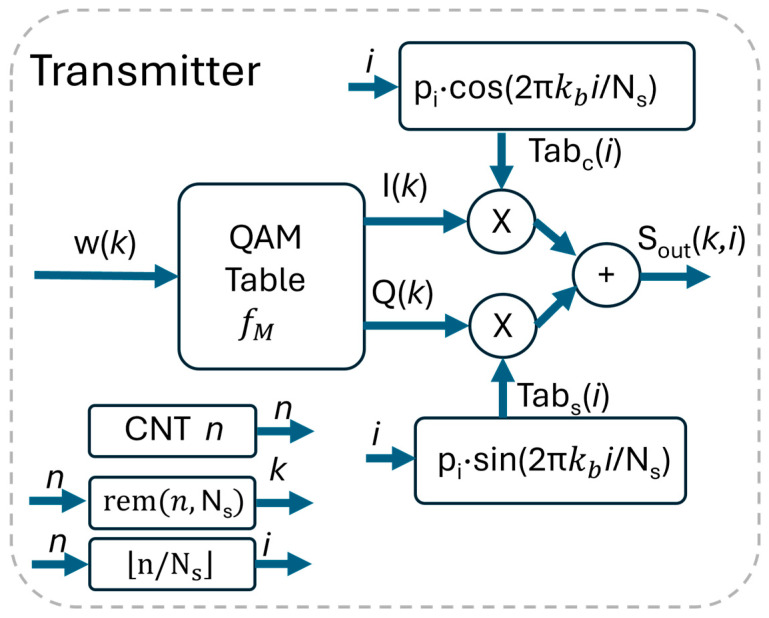
Implementation of the QAM transmitted in FPGA.

The constraints introduced in N. 1 and N. 2 in Table 1 allow the integration of the pulse-shaping and cos/sin functions in the same look-up table and avoid the use of a filter. The realization of the transmitter in the proposed version in FPGA is straightforward. As depicted in Figure 2, it requires just three look-up tables, some trivial sequential logic, in addition to two multipliers and an adder. The first look-up table synthesizes the $f_{M}$ mapping function and has $2^{N}$ entries; the other two tables realize Tab_c_ and Tab_s_ and are composed by $N_{s}$ values. The sequential logic increments the counter *n* every $T_{c}$, so that a new sample is calculated at every new value of the counter. A logic produces the index *i* that addresses the Tab_c_ and Tab_s_ tables by performing $i=\left\lfloor n/N_{s}\right\rfloor ,$ where $\left\lfloor x\right\rfloor$ is the integer part of x. The last sequential logic increments the index *k* every $N_{s}$ steps of *n*, by calculating *k* = rem($n,N_{s}$), where rem(a,b) is $a-\left\lfloor a/b\right\rfloor b$.

### 3.2. The Receiver

**Figure 3:**
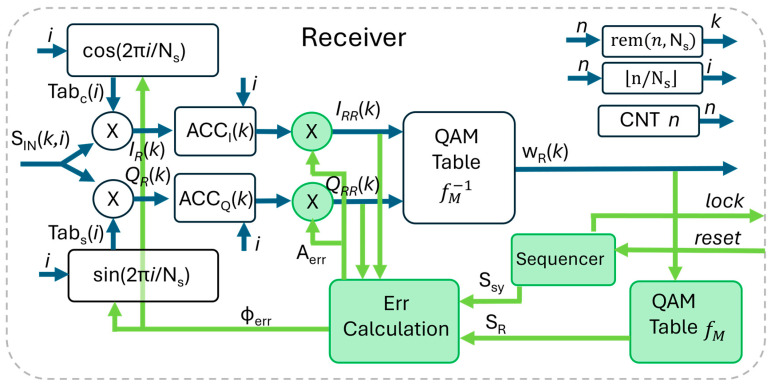
Implementation of the QAM receiver in FPGA. The green blocks and paths refer to synchronization.

The receiver, depicted in Figure 3, is more complex than the transmitter. The blocks in green are related to the synchronization: they will be described later. In this subsection, we assume the receiver is locked: $f_{pt}=f_{pr}=k_{b}/N_{s}$ and Φ=0.

As anticipated in N. 3 in Table 1, we avoid the use of the quadrature demodulator followed by the low-pass filter whose FPGA implementation requires notable resources [32]. The input signal is digitally converted in $S_{IN}\left(k,i\right).$ Then, it is multiplied by the cos/sin function generated by the local oscillator, whose frequency is not the carrier $f_{pt}$ but the ${1/T}_{S}=T_{c}/N_{S}$. The signal sampled at $t=iT_{c}$, after solving the convolution with $p_{T}(t)$ as above, is

(12) $$I_{R}\left(k\right)=G\cdot I\left(k\right)p\left(i\right)cos\left(2\pi \frac{k_{b}}{N_{s}}i\right)cos\left(2\pi \frac{i}{N_{s}}\right)+\;+G\cdot Q(k)p\left(i\right)cos\left(2\pi \frac{i}{N_{s}}\right)sin\left(2\pi \frac{k_{b}}{N_{s}}i\right)\;\;Q_{R}\left(k\right)=G\cdot Q\left(k\right)p\left(i\right)sin\left(2\pi \frac{k_{b}}{N_{s}}i\right)sin\left(2\pi \frac{i}{N_{s}}\right)+\;+G\cdot I(k)p\left(i\right)cos\left(2\pi \frac{k_{b}}{N_{s}}i\right)sin\left(2\pi \frac{i}{N_{s}}\right)$$

We proceed by integrating the above equations in the bit time $T_{s}$, i.e., we perform a summation on the index i for $0\leq i<N_{s}$. The second terms of the additions, where the function sine and cosine are mixed, are

(13) $$G\cdot Q\left(k\right)\sum_{i=0}^{N_{s}-1}p\left(i\right)cos\left(2\pi \frac{i}{N_{s}}\right)sin\left(2\pi \frac{k_{b}}{N_{s}}i\right)=0\;\;G\cdot I\left(k\right)\sum_{i=0}^{N_{s}-1}p\left(i\right)cos\left(2\pi \frac{k_{b}}{N_{s}}i\right)sin\left(2\pi \frac{i}{N_{s}}i\right)=0$$

The summations in (13) are the product of three functions: p(i) and cos(·) are symmetric with respect to the middle of the symbol interval, while $sin\left(\cdot \right)$ is anti-symmetric. In summary their product is anti-symmetric, and the summations (13) are null.

The integration of (12), considering (13), is

(14) $${ACC}_{I}\left(k\right)=G\cdot I\left(k\right)\sum_{i=0}^{N_{s}-1}p\left(i\right)cos\left(2\pi \frac{k_{b}}{N_{s}}i\right)cos\left(2\pi \frac{i}{N_{s}}\right)=G_{I}\cdot I\left(k\right)\;\;{ACC}_{Q}(k)=G\cdot Q(k)\sum_{i=0}^{N_{s}-1}p\left(i\right)sin\left(2\pi \frac{k_{b}}{N_{s}}i\right)sin\left(2\pi \frac{i}{N_{s}}\right)=G_{Q}\cdot Q(k)$$

The two summations in (14) are independent from the symbol and evaluate to two constants that, together with G, are here included in $G_{I}$ and $G_{Q}$.

The output of the accumulators is corrected for amplitude by the correction factor $A_{ERR}$ (more on its calculation in next paragraph), so that at the input of the demodulation table (see Figure 3), we have a copy of the original vectors. Finally, the table recovers the digital words $w_{R}(k)$. The logic that produces the *i*, *k* indexes works like in the transmitter.

### 3.3. The Synchronization

The parameters that should be dynamically tuned are the amplification correction factor $A_{err}$, the phase Φ, and the frequency $f_{pr}$. The synchronization process implemented works in two steps: in the first step, the algorithm recovers the amplitude and the phase through a data-aided process and thus achieves the lock condition; then, the data reception starts, and the algorithm dynamically maintains the correct phase and amplitude with a data-independent process.

We start the description from the second step, when the receiver is locked. With reference to Figure 3, the received symbol $S_{RR}(k)=\left(I_{RR}(k),Q_{RR}(k)\right)$ is converted in the digital word $w_{R}(k)$ in the QAM demodulator $f_{M}^{-1}$ and then is converted back to the vector $S_{R}(k)=\left(I_{R}(k),Q_{R}(k)\right)$ in the QAM map $f_{M}$ present inside the receiver itself. Thanks to this loop, $S_{R}(k)$ represents the ideal point of the constellation corresponding to $S_{RR}(k)$, without noise or phase/amplitude errors. Thus, $S_{R}(k)$ can be compared to $S_{RR}(k)$ to correct possible errors of phase and amplitude. Notably, the phase tracking performed during the lock state inherently compensates for frequency offsets between the transmitter and receiver clocks (as illustrated in the example below).

The aforementioned procedure works only when the lock is achieved, and the errors are low enough not to hamper the correct detection of $S_{R}(k)$. At the onset of communication, the initial phase and amplitude estimates are often arbitrary, and the incoming symbols are not correctly detected. To resolve this, a data-dependent synchronization procedure is employed: at the beginning of the communication, the known symbol $S_{SY}$ is sent. The error calculation block bypasses the potentially erroneous decisions from the receiver’s de-mapper and uses instead $S_{SY}$ in input to the correction process.

In summary, to get the lock, the TX sends a sequence of $S_{SY}$ symbols (e.g., 1000), and the receiver corrects the phase and amplitude using $S_{SY}$ at the input of the “Err Calculation” block. As soon as the phase and amplitude approach the correct value, the RX detects $S_{RR}(k)$ = $S_{SY}$ and uses this condition to switch to the lock state. In the present code, the sequencer visible in Figure 3 moves to the lock condition after 100 consecutive $S_{SY}$ symbols are correctly detected.

A robust strategy for managing the transition between acquisition (non-lock) and tracking (lock) states involves organizing data into discrete packets, each preceded by a synchronization preamble of $S_{SY}$ symbols. In our experimental setup, we utilize a payload of 1 MSymbol prefixed by a 1 kSymbol training sequence. This structure ensures that the receiver re-synchronizes at the start of every packet, preventing long-term drift. A packet-manager (working at higher level with respect to this code) detects the packets and re-initializes the sequencer (reset signal in Figure 3) to trigger a fresh synchronization search for the subsequent preamble.

Below, we present a detailed description of the algorithms employed for amplitude and phase synchronization follows.

#### 3.3.1. Amplitude Correction

The correction factor to be applied in the next symbol, $A_{err}(k+1)$, can be theoretically calculated from $S_{RR}(k)$ and $S_{R}(k)$ (or $S_{SY}$ if the lock condition is not achieved) with

(15) $$A_{err}\left(k+1\right)=\sqrt{\frac{{\left|S_{R}\right|}^{2}}{{\left|S_{RR}\left(k\right)\right|}^{2}}}=\sqrt{\frac{I_{R}^{2}+Q_{R}^{2}}{{I_{RR}}^{2}(k)+{Q_{RR}}^{2}(k)}}$$

In other words, $A_{err}\left(k+1\right)$ is the gain that makes the received symbols match exactly the amplitude of the ideal corresponding vectors. However, in this work, we did not implement (15) directly, since in FPGA, division and square root are demanding operations, and according to the viewpoint of this work, we aim at a simplified approach. We used instead the process described by the pseudo-code reported in Algorithm 1, based on products and summations only.

**Figure 4:**
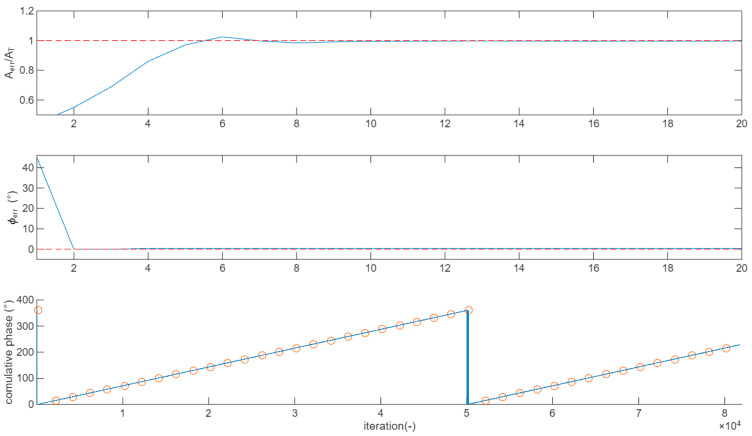
Example of convergence of the correction algorithm for amplitude (**top panel**) and phase (**central panel**). Red dashed line represents the target value. (**Bottom panel**) compares over 80 k symbols the phase tracked by the proposed algorithm (blue line) when the TX and RX frequencies differ by 20 ppm from the theoretical values (red circles).

For every new symbol, the squared amplitudes ${\left|S_{RR}\right|}^{2}$ and ${\left|S_{R}\right|}^{2}$ are calculated (just 2 products and an addition); then, an iteration of the code reported above is executed. The main if clause moves quickly $A_{err}$ to the range 0.5${\left|S_{R}\right|}^{2}$ < ${\left|S_{RR}\right|}^{2}$ < 2${\left|S_{R}\right|}^{2}$. Then, the secondary if structure (in the else branch of the main if) refines the $A_{err}\left(k\right)$ value down to MinRes accuracy. When the gain is approximated at the minimum resolution, the loop acts by tracking the gain with continuous adjustments of ±MinRes. The top panel of Figure 4 reports an example, where $\left|S_{R}\right|=383$, $\left|S_{RR}(1)\right|=310$, and MinRes = 1/1000. The $A_{err}\left(k\right)$ normalized with respect to the goal $A_{T}$ is reported. The goal is achieved in less than 10 iterations after a small overshoot.
**Algorithm 1:** Amplitude Correctionx = 0.25
$A_{err}\left(k\right)=1$ LOOP for each symbol:
    If ${\left|S_{RR}\right|}^{2}$ > $2{\left|S_{R}\right|}^{2}$        $A_{err}\left(k+1\right)\;$= $3/4A_{err}\left(k\right)\;=\;A_{err}\left(k\right)-A_{err}\left(k\right)/4$    Else if ${\left|S_{RR}\right|}^{2}$ < $0.5{\left|S_{R}\right|}^{2}$        $A_{err}\left(k+1\right)\;$= $5/4A_{err}\left(k\right)=\;A_{err}\left(k\right)+A_{err}\left(k\right)/4$    Else       If ${\left|S_{RR}\right|}^{2}$ > ${\left|S_{R}\right|}^{2}$           $A_{err}\left(k+1\right)$ = $A_{err}\left(k\right)$ − x       Else if ${\left|S_{RR}\right|}^{2}$ < ${\left|S_{R}\right|}^{2}$          $A_{err}\left(k+1\right)$ = $A_{err}\left(k\right)$ + x       Else          $A_{err}\left(k+1\right)$ = $A_{err}\left(k\right)$       End       If x > MinRes          x = x/2;       End    End END LOOP

#### 3.3.2. Phase Correction

The Err Calculation block corrects the phase with a similar approach to that employed for the amplitude. The mathematical formula for calculating the phase error between the incoming symbol $S_{RR}(k)=\left(I_{RR}(k),Q_{RR}(k)\right)$ and the reference $S_{R}(k)=\left(I_{R}(k),Q_{R}(k)\right)$ (or $S_{SY}\;$ if the lock is not achieved) is

(16) $$\Phi_{err}={tan}^{-1}\left(I_{R}Q_{RR}-Q_{R}I_{RR},I_{R}I_{RR}+Q_{R}Q_{RR}\right)$$

where ${tan}^{-1}$ is the inverse tangent function. To minimize hardware overhead, we avoid a direct implementation of (16), as the arctangent function is computationally expensive to realize in FPGA logic. Instead, we propose the process based on successive approximation reported in Algorithm 2. In that code $\langle x\rangle$ is the phase of x.
**Algorithm 2:** Phase correctionLOOP for each symbol:    If $\langle S_{RR}\rangle$ and $\langle S_{R}\rangle$ are in different quadrants        $\Phi_{err}(k+1)=\Phi_{err}(k)-\pi /4,or\;\Phi_{err}(k)+\pi /4,\;or\;\Phi_{err}(k)+\pi /2$    Else if $\langle S_{RR}\rangle$ and $\langle S_{R}\rangle$ are in different octants        $\Phi_{err}(k+1)=\Phi_{err}(k)-\pi /8,\;or\;\Phi_{err}(k)+\pi /8$    Else       If $\langle S_{RR}\rangle$ > $\langle S_{R}\rangle$           $\Phi_{err}(k+1)=\Phi_{err}(k)-AngMinRes$       Else           $\Phi_{err}(k+1)=\Phi_{err}(k)+AngMinRes$       End    End END LOOP

The process corrects, first of all, the quadrant, then the octant, and proceeds with steps of AngMinRes, i.e., the angular resolution. In the worst case, considering for example an angular resolution of 0.36°, we need four iterations for getting the right octant and 12.5°/0.36° = 35 iterations to reach the maximum accuracy.

The phase is corrected by acting on the address generation of the cos/sin tables visible in Figure 3. The cos/sin table must have a suitable resolution to accommodate fine phase adjustment. For example, a table with 1000 entries allows AngMinRes = 360°/1000 = 0.36°.

The central panel of Figure 4 reports an example, where $\Phi_{err}$ decreases quicky from 45° to 0.36° in 2 iterations, then the correction proceeds for the next 10 iterations with steps of AngMinRes, to finally reach the goal of $\Phi_{err}<AngMinRes$.

The bottom panel of Figure 4 shows how the proposed phase correction algorithm tracks, during the lock condition, a frequency difference between the TX and RX oscillators of 20 ppm. The blue curve represents the cumulative phase tracked by the algorithm; the red circle reports the phase error calculated from the frequency difference. As expected, a 360° rotation occurs every 50 k symbols.

### 3.4. Performance and Limitations of the Proposed Methodology

This brief subsection is devoted to an evaluation of the performance of the proposed method, with reference to the effects of the simplifications introduced in Table 1.

If *B* is the bandwidth of the channel, the maximum efficiency is obtained with $f_{p}=B/2$. Given the constraint N. 1 in Table 1, the maximum symbol rate is obtained with $k_{b}=1$, i.e., ${{1/T}_{S}=f}_{p}$. In this condition, due to the length limitation of the pulse-shaping function (N. 2 Table 1), the bandwidth of the signal spreads over the whole bandwidth B, and the data rate, related to the constellation points, is limited mainly by ISI. Experiments will show that M = 64 is a good choice for maximizing the data rate. The bandwidth can be reduced by lowering the symbol rate, for example using $k_{b}=2$ (${{1/T}_{S}=f}_{p}/2$). In this case, constellations of higher order can be used, and the limit becomes the SNR.

The maximum data rate in most of the practical conditions is granted by M = 64 and $k_{b}=1$. In fact, to achieve the same data rate with $k_{b}=2,$ we would need M = 4096, which would require a critically high SNR.

## 4. FPGA Implementation

### 4.1. Parameters and Mathematical Limitations

The algorithm was coded entirely in VHDL. The code has as input several parameters (‘generics’ in VHDL) that can be set in compilation that allow tuning the code for different conditions. Table 2 summarizes the parameters and lists the values used in the experiments described in the following part of this work. Given the AD converter rate of $f_{c}=\;$40 MHz, B = 2.7 MHz (see Section 5.2), and FPGA clock $f_{ck}=\;$40 MHz, the parameters tested in the experiments grant the higher transfer rate. The left-most column reports the VHDL parameters, and the second column shows the corresponding value used in the experiments. The third column connects the VHDL parameter to the symbols used in Section 3.

The M parameter, i.e., the constellation points, must obviously be the same for TX and RX, and these are reported in the first section of the table. The other parameters can be different, provided that their combination results in the same $f_{p}$ and $f_{s}$ in the TX and RX sides. The parameter N_b_, present in the TX and RX sections, sets the number of bits of the vectors in the TX and RX constellations. Theoretically, it can be different in TX and RX, but in the experiments, we set it to 10 bits for both. With this value, the vectors are quantized in the range [−512, +512). The number of bits for the Tab_c_/Tab_s_ tables in TX and cos/sin in RX is determined by N_win_ and N_cs_, set to 10 for both. The TABLE_P_ value sets the number of entries in Tab_c_/Tab_s_, and since the table is read at $f_{ck}$, we have in TX, $f_{p}=f_{ck}/$TABLE_P_. In RX, the carrier frequency is set by NCSX, which sets the number of clock cycles for the carrier period. The ‘Data Divisor’ determines the sampling frequency $f_{c}$. It is not a real parameter but a ‘data valid’ input signal used by the driver to select the clock cycles where valid input data are present. In our example, it is fixed to high; so, $f_{c}=f_{ck}$, and the data are sent to the Digital-to-Analog converter and received from the Analog-to-Digital converters at 40 Msps. The CS_TAB_ counts the entries in the cos/sin table in RX and thus determines the angular resolution AngMinRes in the correction of the phase Φ. We set it to 1000. Finally, we have two parameters that are hardcoded: the Hann pulse-shaping function in TX and the AmpMinRes set to 0.1%.

### 4.2. Simulations of the Mathematical Performance

The parameters like, for example, the number of bits of the tables and the constellations, here set to 10 bits, and the resolution for angular and amplitude correction, here set to 0.36° and 0.1%, have been determined by investigating the performance of the algorithm through simulations. The mathematical processing implemented in VHDL has been duplicated in a Matlab^®^ digital twin with care to include all the limitations due to the fixed-point mathematics and the effects of the finite entries of tables. The effect of the frequency offset and different initial phase between TX and RX local oscillators were simulated as well.

Random data packets composed by 1 M of symbols were generated in Matlab^®^. The preamble necessary for synchronization was added to the data packets before they were transmitted and received through the aforementioned digital-twin model. The Symbol Error Rate (SER) was calculated as

(17) $$SER\left\{\begin{array}{c} =\begin{array}{cc} \frac{S_{err}}{S_{tot}} & if\;S_{err}>0 \end{array}\\ \begin{array}{cc} \leq \frac{1}{S_{tot}} & if\;S_{err}=0 \end{array} \end{array}\right.$$

Here, $S_{err}$ is the number of symbols received with errors, and $S_{tot}$ is the number of transmitter symbols for 1 packet ($S_{tot}=1\;M$). The SER values measured with (17) were confirmed on three data packets, which, as shown in [33], grants a confidence level of 95%.

The first test aimed at investigating how the AngMinRes parameter affects the performance. We set the number of bits *N_b_*, *N_win_*, and *N_cs_*, to the very high value of 100, so that they can be considered ideal in the test. Then, we changed CS_TAB_ to change the resolution from 0.18° to 9°. It should be noted that 9° is the lowest resolution attainable with $f_{ck}=\;40\;MHz$ and $f_{p}=\;1\;MHz$, corresponding to CS_TAB_ = 40 =$f_{ck}/f_{p}$. The RX local oscillator was set for a +20 ppm frequency and a +10% phase shift with respect to the TX oscillator.

In the second test, with a similar approach, we investigated how the number of bits affects the performance. In this test, we set AngMinRes to 0.036°, so that it does not interfere with the result, and repeated the test changing the value of *N_b_*, *N_win_*, and *N_cs_*.

**Figure 5:**
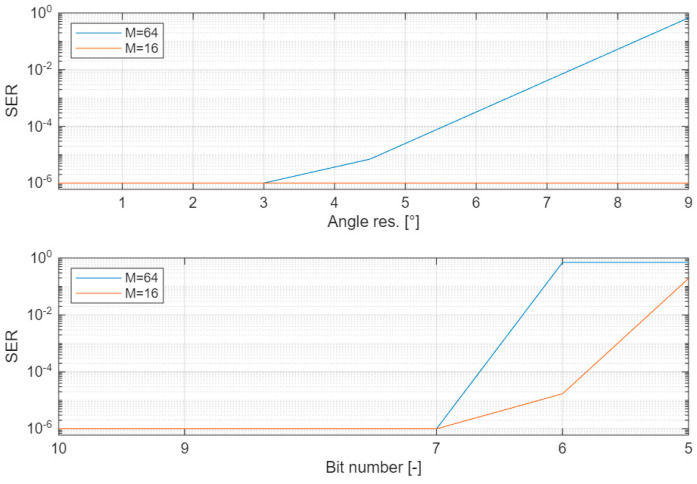
Symbol Error Rate (SER) vs. angular resolution (**top panel**) and vs. number of bits (**bottom panel**). The SER values have a 95% confidence. Blue curve refers to M = 64; red curve refers to M = 16.

The results are reported in Figure 5. The top panel refers to the angular resolution. We note that, for M = 16, we measure SER < 10^−6^ even at the lowest resolution of 9°. For M = 64, we obtain SER < 10^−6^ when the resolution is lower than 3°. The bottom panel reports the results of the investigation about how the number of bits affects the performance. In particular, we report the case where all of the three parameters *N_b_*, *N_win_*, and *N_cs_* assume the same value, variable between 5 and 10. We note that when the number of bits is higher than 7, SER < 10^−6^ is measured for both M = 16 and M = 64.

This analysis confirms that the choice of AngMinRes = 0.36° and *N_b_ = N_win_* = *N_cs_* = 10 listed in Table 2 and employed in the experiments is a conservative choice that grants the maximum performance the algorithm can achieve.

**Figure 6:**
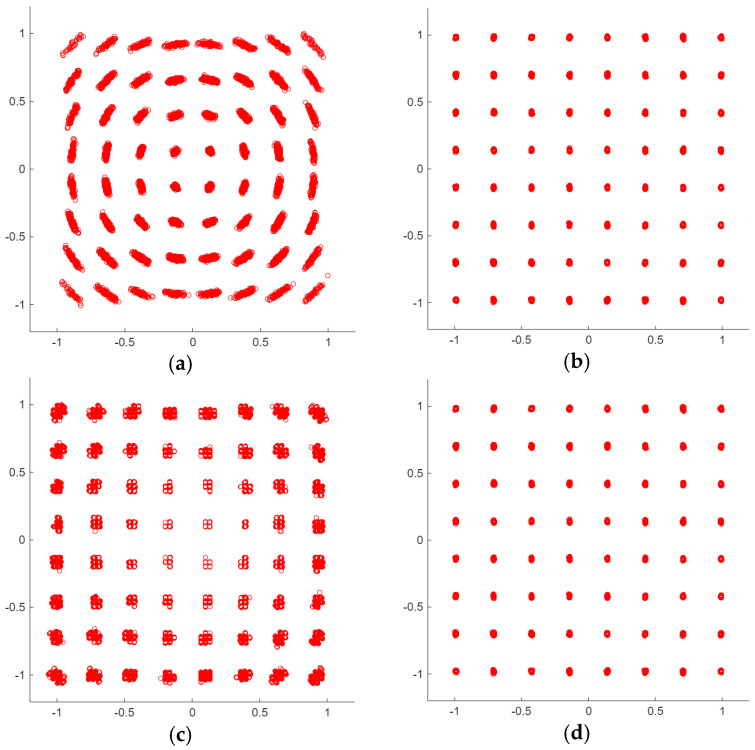
Example of RX constellations elaborated with M = 64 and (**a**): AngMinRes=4.5°; (**b**): AngMinRes=0.36°; (**c**): *N_b_ = N_win_* = *N_cs_* = 7; (**d**): *N_b_ = N_win_* = *N_cs_* = 10.

As a reference, Figure 6 reports four of the constellations measured in the aforementioned tests. In particular, in Figure 6a, the effect of the limited angle resolution is apparent, which produces quantized rotations in the correction of the frequency difference between the TX and RX oscillators. The problem is solved for AngMinRes=0.36°, shown in Figure 6b. A limited number of bits, as shown in Figure 6c, results in a constellation whose points are scattered in nearby quantized positions. Again, this effect is widely reduced for *N_b_ = N_win_* = *N_cs_* = 10, as demonstrated in Figure 6d.

### 4.3. FPGA Resources and Comparison to Other Approaches

We compiled the code on the FPGA 10M50DAF848 from the MAX10 family produced by Intel (Santa Clara, CA, USA), by using the parameters reported in Table 2 and verified in Section 4.2. The resource utilization is listed in Table 3 for M = 16 and M = 64. The resources are separated for the usage of the memory bit implemented through the M9K memory blocks present in Intel MAX10 FPGAs; multipliers implemented from the Digital Signal Processor (DSP) blocks; and Adaptive Logic Modules (ALMs) that realize the standard combinatorial and sequential logics. The usage is further detailed for the modulator, the demodulator, and the synchronization block. These last two blocks represent the receiver.

The compilation confirms that the resource utilization is very low. The modulator, notably, does not require memory, since the small cos/sin table is implemented on ALMs. The demodulator requires more resources. Here, 10 kb of memory (implemented in 2 M9K memory blocks) are used for the sin/cos table, eight multipliers (implemented in a single DSP block) are required for the mathematics, and about 270 ALMs are needed for the logics, 140 of which are implemented with registers. The synchronization block requires two multipliers (1 DSP block) and about 390 ALMs. Notably, expanding the constellation from 16-QAM to 64-QAM requires only a marginal increase in FPGA resource utilization. Even considering the simultaneous implementation of the TX and RX on the same FPGA (right-most columns in Table 3), necessary for example in a full-duplex communication, the total resource utilization is less than 2% for logic and memory and 5% for multipliers when the MODEM is implemented in a 10M50DAF848 device, which behaves as the entry-level MAX10 family. In addition, in a 10M50DAF848, the compilation reaches the time-closure with a clock set at 100 MHz.

The FPGA resources needed by the proposed full QAM TX/RX MODEM are compared to the resources required by the FPGA implementations presented in the related papers analyzed in Section 1.1.

The summary is reported in Table 4. We note that in the implementation reported in [19] (first row of Table 4), the resources are at least one order of magnitude higher with respect to the proposed solution. On the other hand, the project includes OFDM, channel equalization, and data correction. In [20], a M = 4, M = 1024 QAM was realized with channel equalization. Experiments are made by transmitting through an Octavia III taillight and receiving through a PDA36A-EC (Thorlabs, Newton, NJ, USA). Resources are not declared; however, the project is not coded in VHDL but with the use of high-level language tools, whose ease of use is achieved at the expense of efficiency [34]. In [21], resources are not declared as well, and no VLC link is tested. The implementation is limited to the modulator only and is not expected to be significantly less than that proposed. The work [22] reports the implementation of an adaptive equalizer for QAM. This is clearly a computationally intensive block, requiring tens of DSPs. Interestingly, ref. [23] is the only work that focuses on the implementation of the synchronization loop. Two versions are proposed. For both, the ALMs required are comparable to those employed by the proposed implementation (see “synchronization” columns in Table 4); however, the use of DPSs and memory is much higher. No VLC links are tested in this paper. The paper [24] is based on microcontrollers and is of no interest in this comparison.

## 5. Experiments and Results

### 5.1. Experimental Set-Up

**Figure 7:**
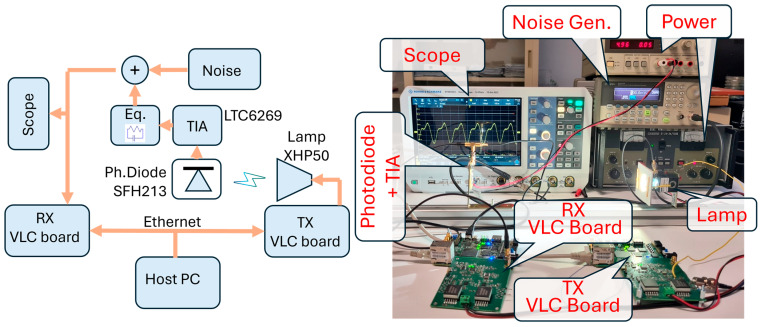
On the right, a photo of the experimental set-up (**right**) is shown, while on the (**left**), a schematic shows the main connections between the employed instrumentations.

The set-up employed in the experiments is shown in Figure 7. Two boards, designed in-house specifically for VLC applications, make possible the real-time implementation of the proposed method. They are two identical boards, one of which is here used for TX (TX VLC board), while the other is for RX (RX VLC board). These boards include a complete TX/RX front-end for VLC and a 10M50DAF848 FPGA that makes possible real-time signal elaboration. The boards are connected via Ethernet to a host PC (not visible in the photo). In the PC, a Matlab^®^ interface allows the user to manage and monitor the boards operations. Interested readers can find a thorough description of the VLC board in [35].

The TX VLC board is connected to the commercial white (5000 K) LED module XHP50 (Cree Inc., Durham, NC, USA). It is actually composed of four LED cells connected in series on the die. It supports a current up to 1.5 A with a voltage drop of about 12 V, for a total power of 20 W. This LED exploits yellow phosphor to generate the white light. The bandwidth of this specific lamp was investigated in the work [36] and resulted in 1.7 MHz when evaluated at −3 dB. The lamp is coupled with a heat dissipator and a short conic reflector.

In the proposed setup, the light is collected by a SFH213 photodetector that drives a house-made Trans-Impedance Amplifier (TIA). The TIA is based on LTC6269 operational amplifier configured for a transimpedance of R = 10 kΩ. A weak post-equalization passive filter was added to extend the bandwidth to 2.7 MHz. A signal generator, model 33250A from Keysight (Santa Rosa, CA, USA), adds a Gaussian noise, whose power is tuned for achieving the desired SNR at the receiver (see below). The resulting signal is conveyed in input to the second VLC board, used as the receiver, and to the RTM3004 scope from Rohde-Schwarz (Berlin, Germany), used for monitoring.

The setup is completed by two bench voltage sources that power the VLC boards and the TIA. Both the TX and RX VLC boards rely on internal DC-DC switching suppliers for generating internal voltages. These kinds of converters are notoriously noisy. In these experiments, we synchronized the switching frequency to the symbol rate. This procedure does not avoid noise [37] but makes the noise the same per every symbol.

### 5.2. Measurements

**Figure 8:**
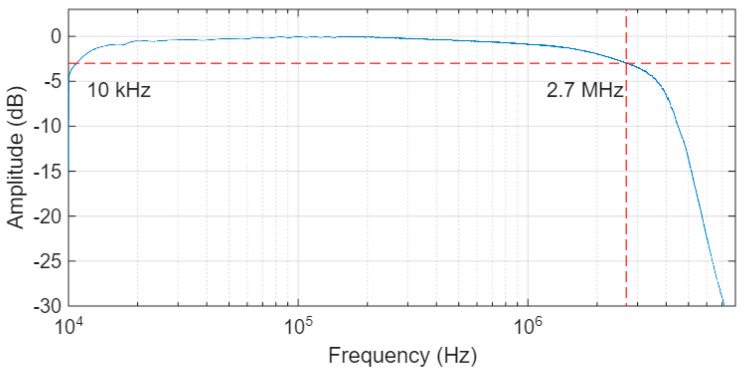
Bandwidth of the full transmission channel measured including the TX board, the lamp, the TIA, the equalizer, and the RX board. Dashed lines highlighted the −3dB bandwidth at 2.7 MHz.

The amplifier integrated in the TX board, which powered the lamp, was set for a DC current of 0.6 A and a modulation index of 50%. A preliminary experiment was conducted to verify the bandwidth of the channel, including the amplifier, lamp, TIA, equalizer, and RX board. The result, illustrated in Figure 8 shows a regular, almost flat, bandwidth that extends between 10 kHz and 2.7 MHz.

A QAM with constellation of M = 4, M = 16, and M = 64 points was used in the experiments. At the symbol rate of 1 M symbol/s, these links communicate at 2 Mb/s, 4 Mb/s, and 6 Mb/s, respectively. The FPGA code presented so far was compiled and downloaded on the FPGA present in the VLC boards.

Using Matlab^®^, random sequences of 1 M symbol were generated with symbols at 2, 4, and 6 bits. These are considered the payload. A preamble composed of 1000 symbols, (1,1) for QAM 04, (3,3) for QAM 16, and (7,7) for QAM 64, was added to the random sequences. The preamble, necessary for the synchronization, represents 0.1% of the payload. The resulting sequence was uploaded to the TX VLC board through Matlab^®^, and the communication was activated. At the end (the transmission lasted about 1 s), the received symbols were downloaded from the RX VLC board to the host PC.

A sequence of experiments was carried out by varying the SNR at the input of the receiver by changing the power of the added noise.

### 5.3. Data Analysis and Comparison to Digital Twin Model

**Figure 9:**
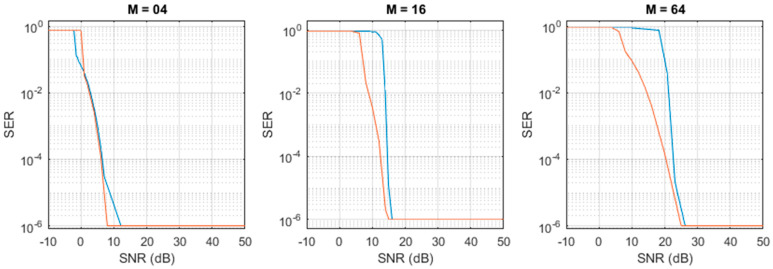
Measured (blue curve) and simulated (orange curve) SER obtained for (**left** to **right**) M = 04, M = 16, and M = 64 (**left**). The SER values have a 95% confidence.

The received symbols were compared in Matlab^®^ to the transmitted sequence, used as ground-truth, and the SER was calculated, as reported in (17). The measured SERs, correlated to the corresponding SNRs, are reported by the blue curves in Figure 9.

The experiment described was repeated in Matlab^®^ by using the digital twin of the proposed method. The digital model was set with exactly the same parameters used in the hardware. As in the experiments, a white Gaussian noise was added as input to the Matlab^®^ receiver to simulate a desired SNR. The SER was calculated and is reported in Figure 9 by the orange curves.

We note a very good agreement between the hardware measurements and simulations when considering the minimum SNR needed for receiving with SER < 10^−6^: 25 dB for M = 64, 15 dB for M = 16, and 10 dB for M = 4. When the SNR decreases, the performance of the hardware measurements reduces a bit more rapidly with respect to the simulation. The discrepancy can be possibly explained by considering the contribution of the non-simulated effects, like, for example, the nonlinearities of the amplifier and the LED, saturations in the electronics, etc.

### 5.4. Latency and Throughput

The proposed FPGA implementation grants a very low latency. In transmission, with reference to Figure 2, the digital word w(k) in input of the QAM table is processed in three pipeline stages: the first is used in the generation of the $I\left(k\right),Q\left(k\right)$ QAM table, the following in the product with ${Tab}_{c}\left(i\right),{Tab}_{s}\left(i\right)$, and the last in the final summation. Considering an extra cycle for the DA converter, the data are present at the LED in four clock cycles: for a clock of $f_{c}$ = 40 MHz, the TX latency sums up to 100 ns only.

In reception, in the hypothesis, the receiver is locked, and with reference to Figure 3, the data in input to the FPGA need a clock cycle to be multiplied with ${Tab}_{c}\left(i\right),{Tab}_{s}\left(i\right)$, $N_{s}$ + 1 cycles for the accumulator (where $N_{s}$ is the symbol temporal length in clock cycles) and two cycles for the amplitude correction and the $f_{M}^{-1}$ table. Considering three clock cycles more for the typical latency of a pipelined AD converter, the overall latency from the LED to the receiver output is $N_{s}+7$ clock cycles. In the reported experiments, where we used $N_{s}$ = 40 and $f_{c}$ = 40 MHz, the receiver latency was as low as 1.175 μs, where $N_{s}/f_{c}$ = 1 μs is the symbol temporal length.

The latency of the proposed implementation is summarized in Table 5. The end-to-end latency (transmitter plus receiver), neglecting the time of flight, accounts to 275 ns more than the symbol time.

**Figure 10:**
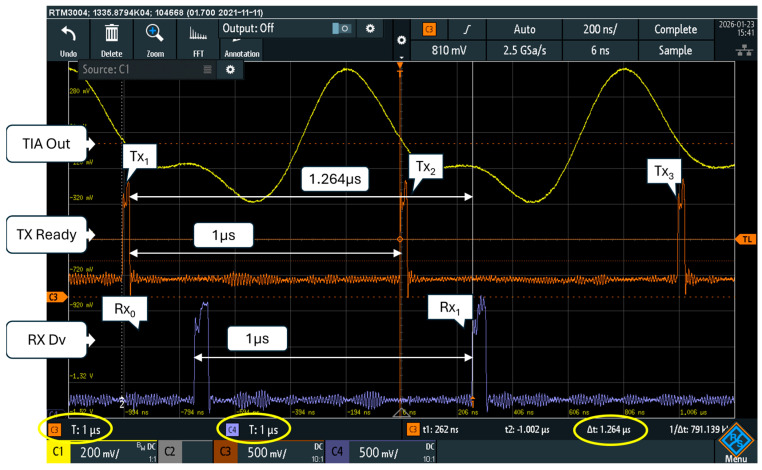
End-to-end latency measured with scope from the ‘Tx Ready’ signal of the modulator (orange trace), and the ‘Rx Dv’ signal in output of the receiver (blue trace). The yellow trace shows the output of the transimpedance amplifier.

Figure 10 shows a scope measurement of the end-to-end latency taken from the “TX Ready” signal that rises to ‘1’ when the modulator accepts a new symbol in input and the “RX Dv” signal that the demodulator activates when the recovered symbol is available on its output. The scope vertical cursors show a 1.264 μs temporal interval, which fits the 1.275 μs shown in Table 5, apart from the tolerances in phase alignment between the transmitter and the receiver. The yellow trace of the scope screenshot shows the output of the transimpedance amplifier.

The proposed MODEM processes a continuous flow of symbols; no pauses among symbols or data-packets are required. In Figure 10, the start of transmissions of the three symbols Tx_1_, Tx_2_, and Tx_3_ is visible, together with the reception of the corresponding symbols Rx_0_ and Rx_1_. Each symbol is transmitted and received every Tb = 1 μs.

## 6. Discussion and Conclusions

In this paper, a very economic and very low latency QAM digital method for FPGA implementation has been proposed. In our opinion, it represents an optimal compromise between resources and performance. The QAM receiver is complete: it includes not only the demodulator but also the logic necessary for the synchronization of the frequency/phase and the correction of the amplitude. In other words, the proposed algorithm is a ready-to-use solution for QAM communications.

The proposed method does not achieve the maximum possible performance attainable in QAM communications. As discussed in Section 3.4, the higher data rate is reached with $f_{p}=B/2$, $k_{b}=1$, and *M* = 64. In this condition, the performance is constrained by ISI. To extend the constellations to orders higher than M = 64, it would be advisable to include a pulse-shaping function of a longer length and more complex algorithms, like channel adaptive equalization [38]. Other improvements include data-independent synchronization [39], spectral clustering [40], and many others. However, all these upgrades would require the use of much higher resources. Moreover, the real-time applications of such complex algorithms may reduce the data rate and increase the latency, as exemplified in the demonstrator [40], where the data rate is limited to 36 symbols per s.

The VHDL code that implements the proposed algorithm is demonstrated for squared constellations; however, by modifying the function that generates the constellation, different point distributions (e.g., circular) can be easily generated.

The code has been implemented in FPGA produced by Intel, compiled through Quartus Prime Lite software. However, the code does not rely on any specific Intellectual Property (IP), is completely written in VHDL, thus is compatible with the free-of-charge compilers available from whichever FPGA vendor, and is distributed in open-source format.

One of the strengths of the proposed algorithm is the inclusion of synchronization. For this implementation, we have chosen a data-dependent method, in agreement with the philosophy behind the project. This method is relatively slow: it needs a sequence of tens of known symbols in the preamble to achieve a lock. But, for the same reason, it is relatively stable: once the lock is achieved, it is maintained even in noisy environments. Several improvements are possible, for example, the addition of filters for further improving its noise immunity or different strategies for increasing the lock velocity.

To summarize, the main advantages of the proposed algorithm are as follows:

- Simplification of the standard approach (based on Table 1) allows a low FPGA use of resources;
- Complete and ready to use, open-source QAM code with modulator, demodulator, and synchronization;
- Flexible parameters (4-16-64-256 QAM, different frequencies, symbol rates, etc.);
- Very low latency.

The limitations and possible improvements are as follows:

- The maximum data rate is limited to $f_{p}=B/2$, $k_{b}=1$, and M = 64 for ISI;
- The addition of channel equalization and/or better anti-ISI filters would improve the performance.

In conclusion, this work proposes a simple, complete, and low-latency QAM method, available for the research community, for real-time VLC experiments. In the authors’ vision, this method can help to fill the void in the current literature of the real-time experimentation of new VLC methods.

## Figures and Tables

**Table 1:** Resource optimization strategies for the proposed QAM implementation.

N.	Brief Description	Applies to
1	Introducing constraints among the symbol rate, the carrier frequency, and the sampling frequency	TX/RX
2	Constraining the length of the pulse-shaping function $p_{T}(t)$ to the symbol time $T_{S}$	TX
3	Substituting the quadrature demodulator tuned at $f_{pr}$ and the following low-pass filter with a demodulator tuned at $1/T_{S}$ followed by an integrator	RX

**Table 2:** Parameters for the customization of the VHDL code.

VHDL Parameter	Value	Parameters for $f_{ck}$ = 40 MHz	Description
Transmitter/Receiver			
M	4, 16, 64	-	Constellation points
Transmitter			
N_b_	10	-	TX constellation number of bits
N_win_	10	-	Number of bits for Tab_c_/Tab_s_ tables
TABLE_P_	40	$f_{p}$	$\mathrm{Number}\;\mathrm{of}\;\mathrm{entries}\;\mathrm{in}\;{\mathrm{Tab}}_{c}/{\mathrm{Tab}}_{s}\;\mathrm{tables},\;\mathrm{corresponding}\;\mathrm{to}\;f_{ck}/f_{p}$
-	-	Hann	Hann pulse-shaping function
Receiver			
N_b_	10	-	RX constellation number of bits
N_cs_	10	-	Number of bits of cos/sin tables
NCSX	40	$f_{p}=f_{ck}/NCSX=1\;MHz$	Carrier frequency
Data Divisor	1	$f_{c}$ = 40 MHz	Sampling frequency
CS_TAB_	1000	AngMinRes = 0.36°	Resolution in phase correction
-	-	*AmpMinRes* = 0.1%	Resolution in amplitude correction

**Table 3:** Resources of MAX10 10M50DAF848 FPGA.

	Modulator		Demodulator		Synchronization		TOT	
M-QAM	16	64	16	64	16	64	16	64
ALM	141	147	269	276	390	395	800	818
Reg	57	61	140	131	136	138	276	330
Multipliers (DSP)	4 (2)	4 (2)	8 (4)	8 (4)	2 (1)	2 (1)	14 (7)	14 (7)
Memory bit (M9K)	0	0	10 kb (2)	10 kb (2)	-	-	10 kb (2)	10 kb (2)

**Table 4:** FPGA resource usage comparison.

Description	FPGA Resources	Target Rate	Paper Ref.
HW: Intel Arria 10 SOC + AD-FMCDAQ2-EBZ 256-QAM OFDM receiver, 32-bit architecture with channel equalization and data correction	TX: ALM → 26,313; RAM → 18,191; DSP → 70 RX: ALM → 19,139; RAM → 45,654; DSP → 48	1 Gb/s	[19]
HW: NI USRP 2921 SDR + PDA36A-EC 4–1024 QAM with equalization realized through high-level synthesis	Resources are not declared	-	[20]
HW: Intel EP2C20F484C7 Fixed 16-QAM, modulator only	Resources are not declared	-	[21]
HW: Xilinx Virtex 4 XC4VSX55 Adaptive equalizer for 16–256 QAM receiver	ALM → 9488; DSP → 74; RAM → not declared	6.9 MS/s	[22]
HW: Xilinx Virtex-II Carrier synchronization block for 16 QAM	Memory-based version: ALM → 254; DSP → 16; RAM → 6 blocks CORDIC-based version: ALM → 413; DSP → 8; RAM → 1 blocks	90 Mb/s	[23]
HW: STM32F4 16-QAM receiver	Microcontroller-based	40 kb/s	[24]

**Table 5:** Latency of the proposed FPGA implementation.

	Latency in CLK	$\mathrm{Latency}\;\mathrm{for}\;f_{c}$$\;=\;40\;\mathrm{MHz}\;\mathrm{and}\;N_{s}$ = 40
TX	4 CLK	100 ns
RX	$N_{s}$+ 7 CLK	1175 ns
TOT	$N_{s}$+ 11 CLK	1275 ns

## Data Availability

The raw data supporting the conclusions of this article will be made available by the authors on request.

## Supplementary Materials

The VHDL code of the QAM MODEM can be downloaded at https://doi.org/10.5281/zenodo.18221551 (accessed on 25 January 2026).
